# Supplementary material for: The podocin V260E mutation predicts steroid resistant nephrotic syndrome in black South African children with focal segmental glomerulosclerosis
Source: Commun Biol. 2019 Nov 15;2:416. doi: 10.1038/s42003-019-0658-1 (PMC6858321; doi:10.1038/s42003-019-0658-1)
Supplement: Supplementary file 1 — Supplementary Information [file 42003_2019_658_MOESM1_ESM.docx]

**Supplementary information**


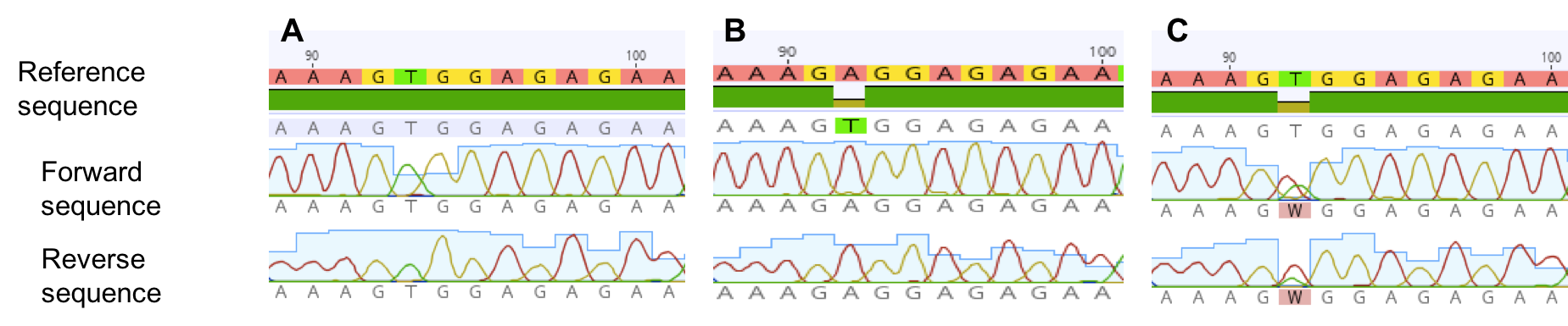
**Supplementary Figure 1:** Electropherograms showing sequence data from individuals (A) Homozygous for the *NPHS2* V260 allele (T/T) (B) Homozygous for the *NPHS2* 260E allele (A/A) (C) Heterozygous for the *NPHS2* V260E allele (A/T), from the sequencing in the forward and reverse directions.

**Supplementary Table 1:** *APOL1* genotype distribution among FSGS cases and controls

| **Genotype**  **Frequency** | **Allele Frequency** | **FSGS (30)** | **SSNS (10)** | **SRNS (20)*** | **Controls (n=176)** |
| --- | --- | --- | --- | --- | --- |
| **0 risk allele**  G0/G0 |  | 14 (46%) | 3 (30%) | 11 (55%) | 117 (66.5%) |
| **1 risk allele**  G0/G1  G0/G2  **Total** |  | 5 (17%)  8 (27%  **13 (44%)** | 3 (30%)  4 (40%)  **7 (70%)** | 2 (10%)  4 (20%)  **6 (30%)** | 20 (11.4%)  27 (15.3%)  **47 (26.7%)** |
| **2 risk alleles**  G1/G1  G1/G2  G2/G2  **Total** |  | 0  1 (3%)  2 (7%)  **3 (10%)** | 0  0  0  **0** | 0  1 (5%)  2 (10%)  **3 (15%)** | 2 (1.1%)  3 (1.7%)  7 (4%)  **12 (6.8%)** |
|  | G0 allele  G1 allele  G2 allele | 0.683  0.100  0.216 | 0.650  0.150  0.200 | 0.700  0.075  0.225 | 0.798  0.077  0.125 |

* Of the 20 SRNS 20 cases only 5 cases did not have the V260E variant (3 of these individuals had the G0/G0 genotype, 1 individual had G0/G2 genotype and 1 individual had G2/G2 genotype)
